# A framework to assess mechano-tribological, structural and biochemical properties of articular cartilage

**DOI:** 10.3389/fbioe.2026.1945270

**Published:** 2026-10-01

**Authors:** Manoj Rajankunte Mahadeshwara, Hemant Pandit, Maisoon Al-Jawad, Richard M. Hall, Michael Bryant, Reem El-Gendy

**Affiliations:** 1 Institute of Functional Surfaces, Mechanical Engineering, University of Leeds, Leeds, United Kingdom; 2 Department of Oral Biology, Faculty of Dentistry, University of Leeds, Leeds, United Kingdom; 3 Leeds Institute of Rheumatic and Musculoskeletal Medicine (LIRMM), University of Leeds, Leeds, United Kingdom; 4 School of Engineering, College of Engineering and Physical Sciences, University of Birmingham, Birmingham, United Kingdom; 5 Department of Oral Pathology, Faculty of Dentistry, Suez Canal University, Ismailia, Egypt

**Keywords:** articular cartilage, boundary lubrication, collagen organization, microscale mechanics, microscale tribology, Raman spectroscopy

## Abstract

**Introduction:**

Articular cartilage (AC) is a specialized tissue with a unique biphasic structure that enables the high load-bearing ability and near-frictionless joint motion. The macroscale mechanical and tribological behavior of AC is dominated by interstitial fluid pressurization. Hence, microscale studies provide an opportunity to characterise local mechanical, tribological, structural and biochemical properties that may otherwise be limited during macroscale evaluation.

**Methods:**

This study investigates an integrated assessment of the microscale surface properties of bovine AC by combining microscale indentation and reciprocating sliding experiments alongside Raman spectroscopy, scanning electron microscopy, and histomorphometry.

**Results:**

Microscale mechanical testing revealed a uniform effective instantaneous modulus at the surface with varying load (ranging 0.43–0.51 MPa, p = 0.452) and varying indentation rate (ranging 0.38–0.46 MPa, p = 0.621), along with compressive strain dependent viscoelastic behavior. Tribological testing revealed low microscale coefficients of friction with varying load (ranging 0.001–0.036, p = 0.005) and varying speed (ranging 0.002–0.047, p = 0.012). Further, zonal characterization showed uniquely arranged ECM orientation patterns across superficial, middle, and deep zones alongside depth-dependent variations in cellular attributes (cellular density, orientation, and proteoglycan intensity) and biochemical constituents (collagen organisation and proteoglycan distribution).

**Discussion:**

Collectively, these findings highlight the feasibility of combining microscale mechano-tribological testing with structural and biochemical characterisation to assess local properties of AC. The framework demonstrates load and rate-dependent functional behaviour at the surface and cross-sectional variations in cartilage structure and composition that are often dominated by interstitial fluid pressurisation during macroscale evaluation.

## 1 Introduction

AC provides structural and functional support for diarthrodial joints and degeneration of AC leads to osteoarthritis (Simick Behera et al., 2024). Osteoarthritis is a chronic disorder which progresses irreversibly and has affected 595 million individuals globally, accounting for roughly 7.6% of the entire world population (Wang et al., 2025). This imposes a major health and economic burden, accounting for 1%–2.5% of healthcare expenditure in high-income countries (Cao et al., 2024). The interaction between the structure and function of AC is pivotal in determining the degradation patterns observed in osteoarthritis (Rajankunte Mahadeshwara et al., 2024). Therefore, it is essential to explore how these characteristics are interconnected and mutually dependent. AC tissue (typically 2–6 mm thickness for bovine cartilage) consists of ∼80% of water and ∼20% of solid ECM and presents two primary structural characteristics, relating to ECM organization and depth dependency. This structural organisation governs overall cartilage function (Figure 1) (Sophia Fox et al., 2009).

**FIGURE 1:**
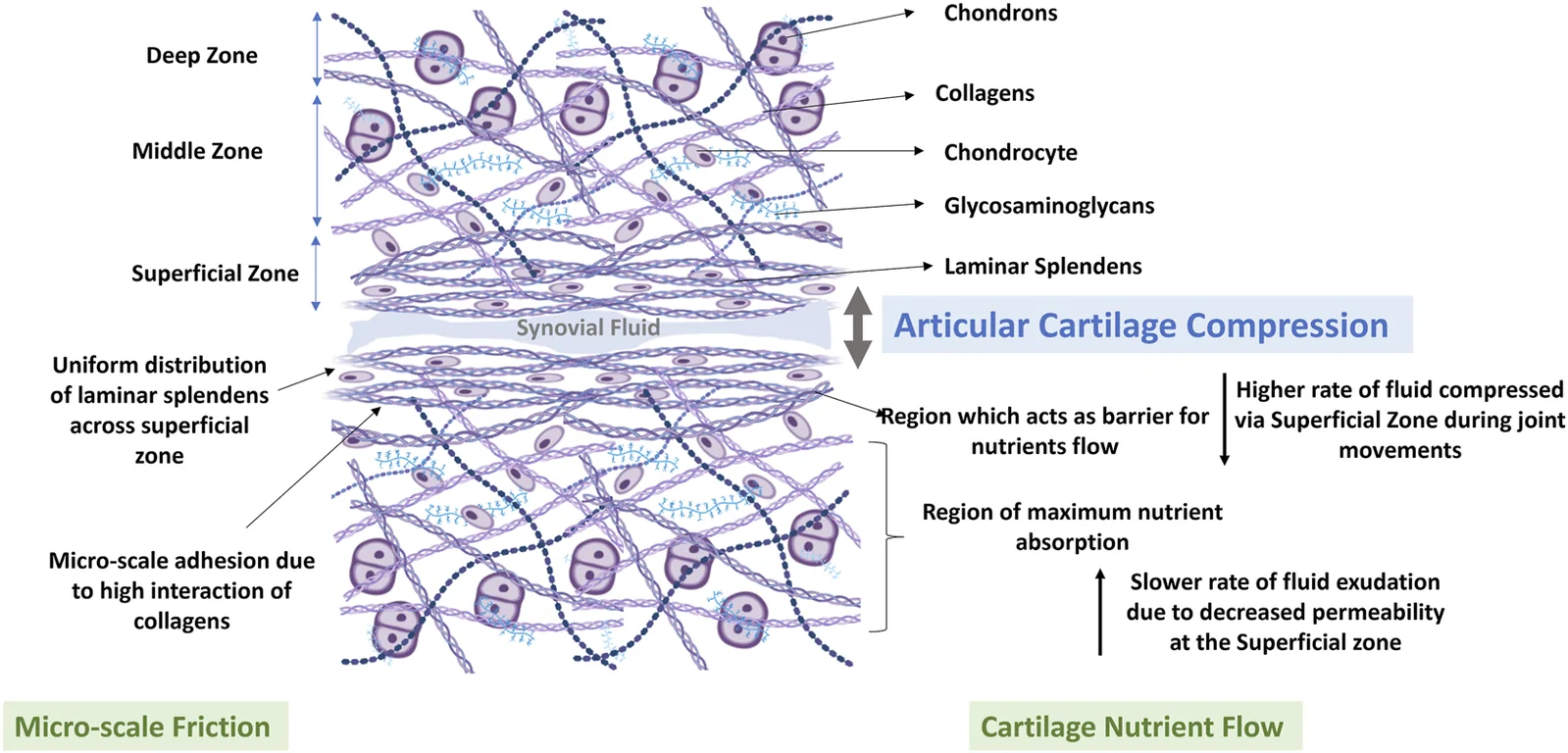
The schematic representation of the articular cartilage compression showing the visualisation of the factors affecting the microscale friction and the nutrient flow across the different zones of the cartilage matrix.

Established mechanical and tribological tests on AC such as confined or unconfined compression, bulk indentation studies have investigated fluid-supported load-bearing behaviour. However, the contribution of the solid ECM is largely ignored (Mansour, 2003; Halloran et al., 2012). Therefore, microscale studies are required to minimise interstitial fluid pressurisation to understand the ECM stiffness directly (Halloran et al., 2012). At the macroscale, up to 90% of compressive load or physiological loading is supported via fluid pressurisation. Over time this fluid pressure dissipates through flow thereby shifting the load to solid matrix (Park et al., 2003; Ateshian and Wang, 1995). In contrast at the microscale due to shorter lengths and extended loading, the fluid pressurisation decays much faster allowing the moduli to reflect predominantly the solid phase response (Soltz and Ateshian, 1998). Further, microscale studies helps to isolate the local ECM behaviours by considering the ECM fibril orientation, glycosaminoglycan content and collagen crosslinking (Wang et al., 2019). These local heterogeneities are often masked in macroscale studies which plays an important role in understanding the disease initiation and progression of osteoarthritis (Desrochers et al., 2013). Furthermore, these methods also capture the difference between the fluid-film and boundary lubrication thereby providing a platform to study the lubricant-based therapies to improve AC function in osteoarthritis (Yao et al., 2025; Fragassi et al., 2024).

In this study, we have investigated the mechano-tribological behaviour of the bovine AC using microscale indentation and reciprocating sliding experiments. These properties were complemented with qualitative and quantitative characterization of the surface via Raman spectroscopy, scanning electron microscope (SEM) and histomorphometry. Finally, integrating these characterisation and experimental techniques, the study establishes a framework for assessing mechanical, tribological, structural and biochemical characteristics of AC at the microscale.

## 2 Methods

### 2.1 Sample preparation

AC samples were obtained from four bovine femoral condyles (N = 4) with three technical replicates (n = 3) performed for each sample sourced from John Penny and Sons, Leeds, United Kingdom. Osteochondral plugs (8 mm diameter) were extracted from the medial region of each stifle joint using high-speed rotary tool under continuous supply of phosphate-buffered saline. Samples were divided into two groups for surface and zonal analysis. Samples with a diameter of 8 mm were used for surface studies and the samples were sliced into cross-sections with a thickness of 2–3 mm using the Struers Accutom cutter. All samples, except those designated for histological analysis, were preserved at −20 °C and thawed for 4 h prior to testing. Similarly, the histology samples were stored in formalin for further processing.

### 2.2 Microscale mechanical and tribological testing

#### 2.2.1 Microscale indentation testing

Microscale indentation and sliding experiments were performed using a custom-built tribometer equipped with an Anton Paar cantilever mounted on a piezoelectric stage (Normal force stiffness (K_ns_) = 0.4808 mN/µm and tangential force stiffness (K_ts_) = 1.1399 mN/µm). Further, capacitance sensors were used to measure cantilever deflection to determine normal (Fn) and tangential (Ft) forces, while a linear variable differential transformer was used for sample positioning. Figure 2 shows the image of the custom-built micro-tribometer, highlighting its key components and structural configuration.

**FIGURE 2:**
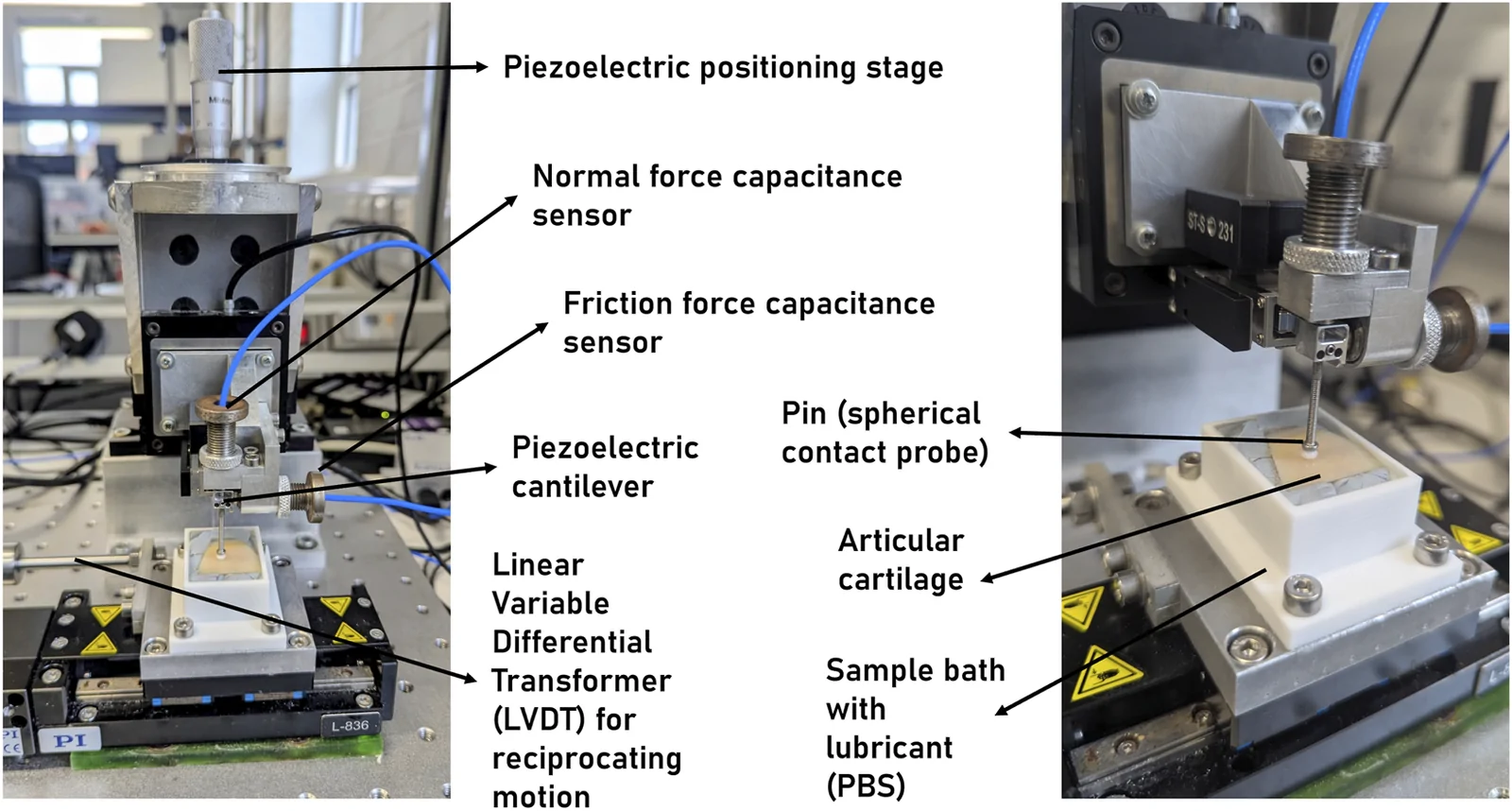
Schematic representation of the custom-built micro-tribometer, highlighting its key components and structural configuration.

Indentation experiments were performed using a 3 mm diameter spherical ceramic indenter (radius = 1.5 mm) mounted on the cantilever by applying prescribed load followed by a holding period and unloading phase to obtain creep responses of the cartilage surface. The cartilage was assumed to be isotropic and nearly incompressible with an assumed Poisson’s ratio of 0.5, consistent with previous microscale indentation studies (Schätti et al., 2016). Mechanical parameters were calculated at loading and holding regions of the indentation curve. The effective instantaneous modulus was determined using Equation 1, compressive strain and creep rate constant were calculated using Equations 2, 3 respectively.

$$F_{n}=\frac{4}{3}E_{r}R^{\frac{1}{2}}{\left(\delta -\delta_{0}\right)}^{3/2}$$ (1)

The Hertzian contact equation relates F_n_ to the indentation depth and material properties (Schätti et al., 2016). In this equation, F_n_ is normal load (N), E_r_ is effective instantaneous modulus (MPa), R is radius of indenter (mm), δ is the indenter displacement (mm) and δ_0_ is the initial displacement (mm) offset given by the software. The elastic modulus of the ceramic indenter was assumed to be much greater than that of cartilage; therefore, indenter deformation was neglected in the Hertz contact analysis. The Hertz contact model was fitted to the loading portion of the force–displacement curve using nonlinear least-squares regression (OriginPro, OriginLab, USA) following initial contact detection and before the holding phase. The maximum indentation depth ranged from 0.014 to 0.042 mm, corresponding to an estimated Hertz contact radius of approximately 140–250 µm.

$$\text{compressive strain }\left(\%\right)=\frac{\Delta h}{h}\times 100$$ (2)

$$\varepsilon =M\left(1-e^{-\text{Tt}}\right)$$ (3)

The compressive strain was calculated as the percentage change in cartilage thickness relative to the original thickness. The empirical Kelvin-Voigt viscoelastic two-parameter exponential model (Mow and Huiskes, 2005; Cutcliffe et al., 2020) was used to fit the strain versus holding time to calculate the creep rate constant (s^-1^). In these equations, ε is the strain, h is the original cartilage thickness (mm) Δh change in cartilage thickness (mm), t is the holding time (s), Τ is the creep rate constant (s^-1^), M is the fitting constant. The exponential fitting parameter T quantifies the rate at which compressive strain approaches equilibrium during the creep response. Higher values of the creep rate constant indicate a faster approach to equilibrium deformation.

#### 2.2.2 Microscale sliding tribology testing

Reciprocating sliding tests were conducted using a custom-built tribometer on an 8 mm AC plug using a 3 mm diameter spherical ceramic probe immersed in phosphate-buffered saline. Normal load (F_n_) was applied through piezoelectric cantilever while tangential force (F_t_) and displacement were recorded during reciprocating motion of the sample stage. Two experimental conditions were investigated with constant load and varying speed and while maintaining a constant speed with varying the load (sliding distance = 0.5 mm). The raw data were acquired using national instruments LabVIEW software with changes in F_n_, F_t_, displacement and from which the COF was calculated using Equation 4. Steady-state COF values were determined from the middle 20% of each reciprocating cycle after application of a moving-average filter in MATLAB, thereby minimising the influence of stroke reversal and transient effects.

$$\text{COF}=\frac{F_{t}}{F_{n}}$$ (4)

### 2.3 Structural and biochemical characterization

#### 2.3.1 Scanning electron microscopy

AC cross-sections (2–3 mm thickness this includes cartilage with subchondral bone attached) were imaged using Hitachi S-3400N operating under variable-pressure conditions. Samples were maintained at a sublimation temperature of −20 °C and a saturated water vapor pressure of 100 Pa to preserve tissue hydration during imaging. Secondary electron images of the full cross-section were initially acquired at ×50 magnification with a 10 kV accelerating voltage, followed by imaging of the superficial (SZ), middle (MZ), and deep (DZ) zones at 1,000× magnification.

#### 2.3.2 Raman spectroscopy

The biochemical composition of AC cross-sections was analysed using Renishaw Raman spectroscopic system. Line scans were performed at ×50 magnification using a 785 nm laser source with 1 mW power. Raman bands corresponding to collagen and proteoglycan constituents were identified from literature and are summarised in Table 1. Spectral data were processed in MATLAB using baseline correction and normalization, followed by peak identification. Further, compositions of AC constituents were measured using the ratios of the normalised intensity values of Amide III random coil to α-helix (I-1241/I-1269) for collagen organisation. While the distribution of proteoglycans was evaluated using SO^3-^ to Amide I ratio (I-1062/I-1670).

**TABLE 1:** The assignments of articular cartilage Raman peaks (Rieppo et al., 2017).

Raman wavenumber (cm^-1^)	Assignment	Collagen and proteoglycan
816	^v^(C-C) Protein backbone	Collagen
856	^v^(C-C) Proline	
875	^v^(C-C) Hydroxyproline	
920	^v^(C-C) Hydroypro/Proline	
943	CCO	​
1,005	^v^(C-C) Phenylalanine	Phe
1,068	SO_3_^−^ stretching of sulfates in glycosaminoglycans	Glycosaminoglycans
1,123	Hyaluronic acid	
1,241	NH_2_ bending: random coil (Amide III)	Collagen
1,269	NH_2_ bending: α-helix (Amide III)	
1,343	CH, glycosaminoglycans	Glycosaminoglycans
1,380	COO^−^, glycosaminoglycans	
1,450	CH_2_/CH_3_ scissoring; collagen and other proteins	Collagen
1,670	C=O stretching, α-helix Amide I	

#### 2.3.3 Histology and histomorphometric analysis

AC tissues were fixed in 4% neutral buffered formalin (Sigma-Aldrich Company Ltd.) for 7 days at room temperature. Followed by decalcification in 12.5% (w/v) ethylenediaminetetraacetic acid (pH 7.4) at room temperature for 4 weeks with solution replacement every 72 h. Further, tissues were dehydrated through graded ethanol concentrations (80%, 90%, and 100%), cleared in xylene and infiltrated with molten paraffin using an automated tissue processor (Leica TP1020, Leica Biosystems, Germany). Samples were embedded in paraffin blocks and sectioned at 8 µm thickness using a rotary microtome before mounting onto glass slides.

Sections were deparaffinized in xylene, rehydrated through descending ethanol concentrations (100%, 90%, and 80%), and stained with hematoxylin and eosin (H&E) and safranin O/fast green (SO & FG) according to established protocols (Schmitz et al., 2010). Following staining, sections were dehydrated in ascending ethanol concentration cleared in xylene and mounted using a permanent mounting medium and coverslip (El-Gendy et al., 2021; El-Sayed et al., 2020).

Histological sections were imaged using a light Nikon microscope and preprocessed using Nikon Elements System software (Version 3 Tokyo, Japan). Images were acquired at ×40 magnification from representative regions containing SZ, MZ, and DZ. Three random fields in each of three sections of four bovine samples were analysed. Quantitative analysis was performed using ImageJ software to determine proteoglycan intensity, cellular orientation, and chondrocyte density (Pastoureau et al., 2003). Proteoglycan intensity was quantified from red-stained regions using inverse mean grayscale intensity, cellular orientation was determined from chondrocyte nuclear alignment relative to the cartilage surface, and cell density was calculated as the number of nuclei within a defined cartilage area.

### 2.4 Statistical analysis

Statistical analyses were performed in MATLAB R2024a, with the significance defined as p < 0.05. Experiments were conducted using a sample size of N = 4 independent samples with technical repetitions of n = 3 per sample unless otherwise stated. The technical replicates were averaged prior to statistical testing for all the biological replicates as one unit for the analysis. The mean, standard deviation, and coefficient of variation (CV = standard deviation/mean × 100%) for the effective indentation modulus, COF, histomorphometry, and Raman spectroscopy datasets were calculated. The normality of the data sets was assessed using Lilliefors test (α = 0.05), if the data were normally distributed, then parametric tests like one-way analysis of variance (ANOVA) was performed and otherwise, the non-parametric Kruskal–Wallis test was used. If an overall significant effect was observed, for parametric Tukey-Kramer *post hoc* multiple comparison tests and for non-parametric Bonferroni-adjusted comparisons were used to identify pairwise differences.

## 3 Results

The microscale mechanical properties of the AC were evaluated using the effective instantaneous modulus, compressive strain and creep rate constant. Similarly, tribological properties were evaluated based on COF values. Surface and zonal characterization were further performed to assess the structural and compositional organization of the ECM across cartilage zones.

### 3.1 Microscale mechanical properties of articular cartilage

Microscale indentation experiments at the AC surface were performed varying load conditions at constant indentation rate (Figure 3A) and varying the indentation rates at constant load (Figure 3B). The graphs showing the loading region curve fits are represented in Figures 3C,D. For varying load (Figure 3E), the effective instantaneous modulus ranged from 0.43 ± 0.06 to 0.51 ± 0.09 MPa with CV values ranging from 6.39% to 24.03% with no significant differences observed between groups (One-way ANOVA, p = 0.452). However, for varying indentation rate (Figure 3F), the effective instantaneous modulus ranged 0.38 ± 0.02 to 0.46 ± 0.11 MPa with CV values ranging from 15.33% to 23.52% with no significance on modulus (One-way ANOVA, p = 0.621).

**FIGURE 3:**
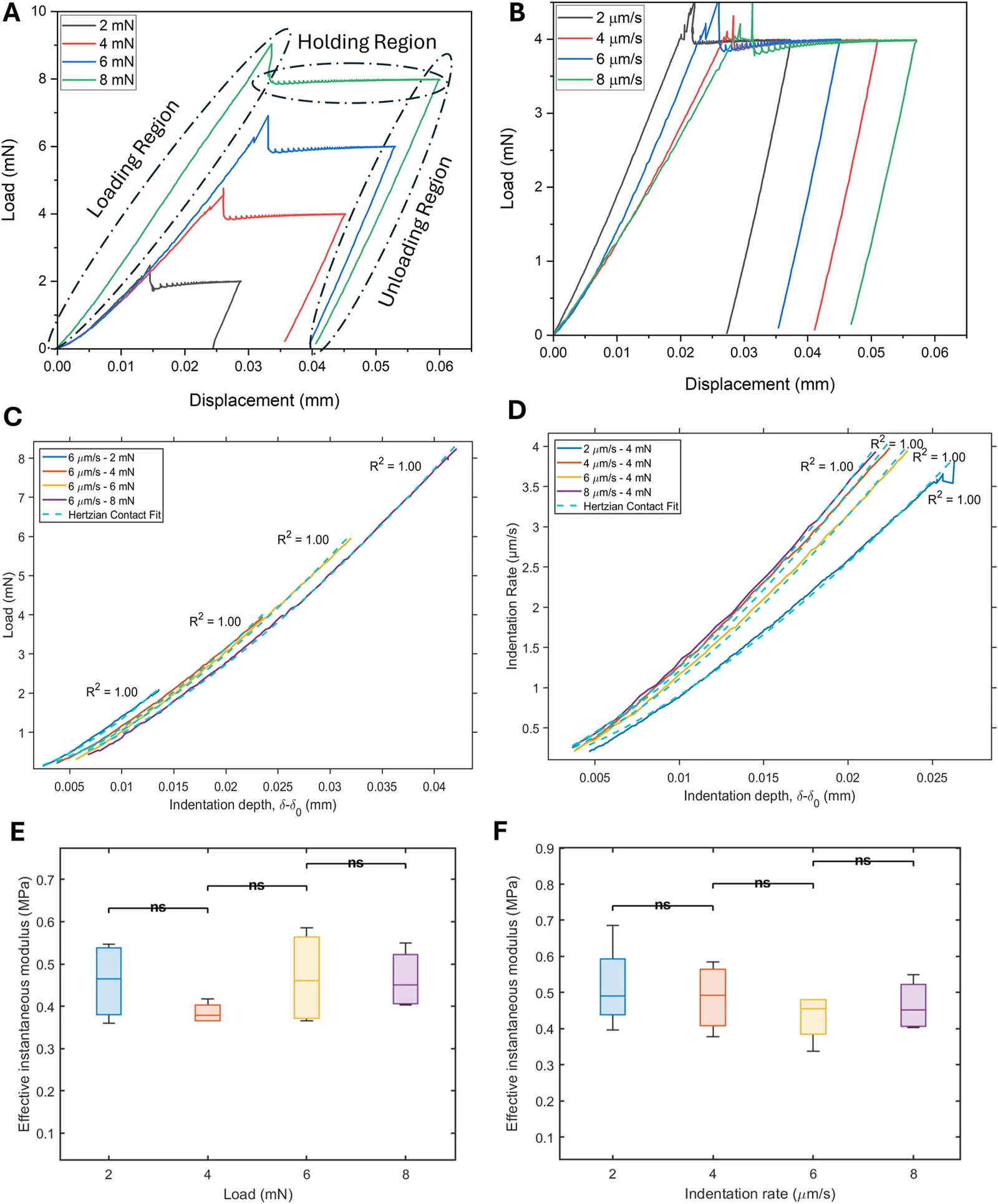
The plot of indentation data showing the **(A)** load v/s displacement with varying load and constant indentation rate (6 µm/s). **(B)** load v/s displacement with varying indentation rate and constant load (4 mN). The graphs showing the loading region of the indentation curve with curve fitting using the Hertzian contact equation to calculate the effective instantaneous modulus **(C)** with constant indentation rate and change in load **(D)** with constant load and change in indentation rate. **(E)** The variation in effective instantaneous modulus with applied load with indentation rate constant (6 µm/s). **(F)** The variation in effective instantaneous modulus with indentation rate with load constant (4 mN). Data are presented as box plots showing the median, interquartile range, and range. No significant differences were observed between groups (one-way ANOVA, p > 0.05; ns = not significant).

Compressive strain and creep rate constant were evaluated under varying load and indentation rate conditions (Figures 4A,B). For varying load (Figure 4C), the compressive strain ranged 1.12% ± 0.42% to 2.18% ± 0.40% with CV values ranging from 3.31% to 40.38% with no significant difference (Kruskal-Wallis, p = 0.23). Similarly (Figure 4D), the creep rate constant ranged 0.03 ± 0.01 to 0.05 ± 0.002 s^-1^ with CV values ranging from 46.57% to 111.61% with no significant effect (Kruskal-Wallis, p = 0.55). Under varying indentation rates the compressive strain was significant with p = 0.023 (Kruskal-Wallis) (Figure 4E) and ranged 1.34% ± 0.39% to 1.88% ± 0.63% with CV values ranging 2.33%–16.61% although pairwise comparisons did not reveal significant differences between individual groups. In contrast, the creep rate constant was not significant with indentation rate (Kruskal-Wallis, p = 0.247) and ranged 0.02 ± 0.01 to 0.04 ± 0.01 s^-1^ and CV 4.72%–56.51% (Figure 4F).

**FIGURE 4:**
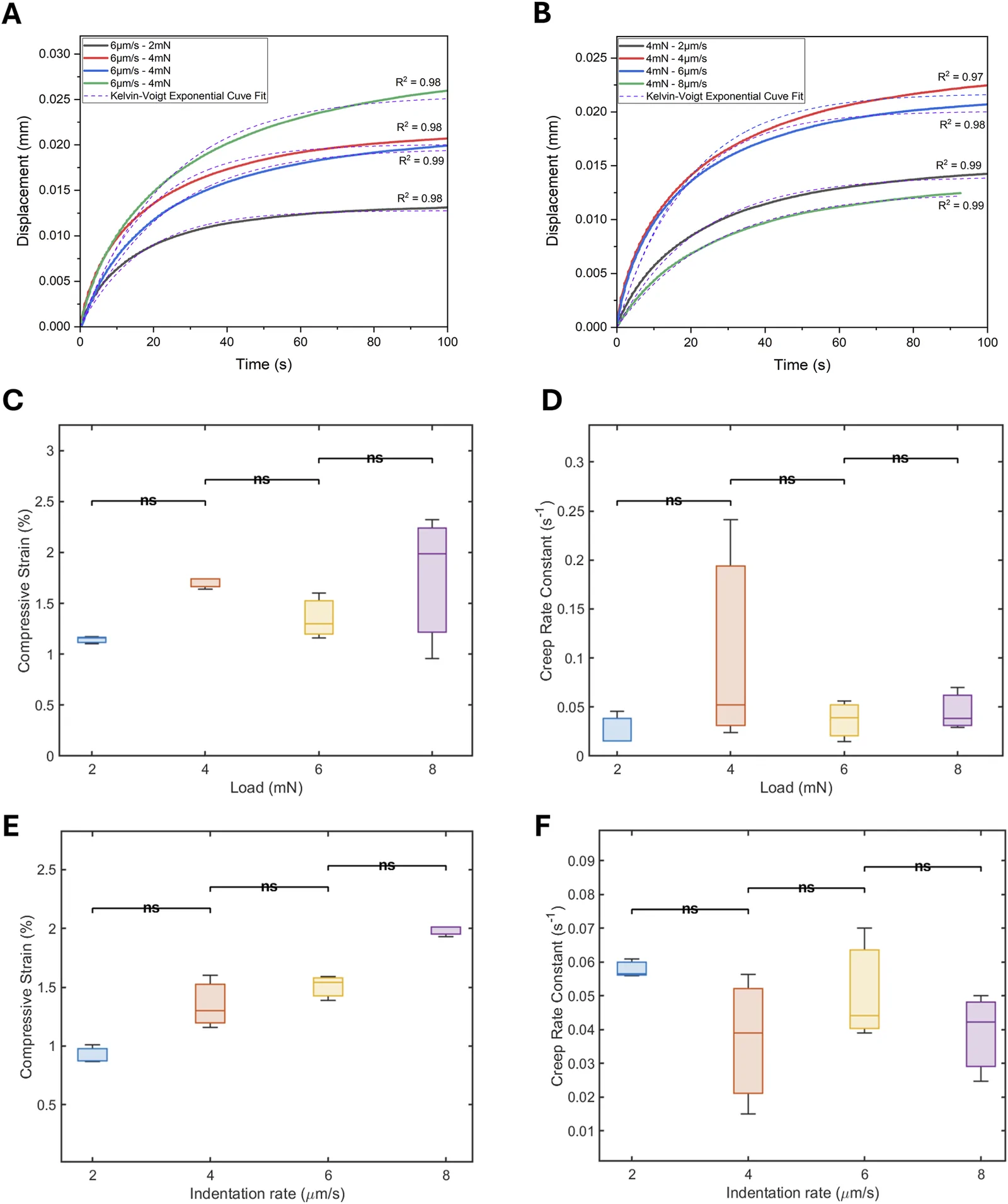
The graphs showing the holding region fitted with Kelvin Voigt exponential curve fit to calculate the creep rate constant for the curves **(A)** with change in load and constant indentation rate **(B)** with change in indentation rate and constant load. **(C)** The variation in compressive strain as a function of applied load with indentation rate constant (6 µm/s). **(D)** The variation of creep rate constant as a function of applied load with indentation rate constant (6 µm/s). **(E)** The variation of compressive strain as a function of indentation rate with load constant (4 mN). **(F)** The variation of creep rate constant as a function of indentation rate with load constant (4 mN). No significant differences were observed with varying load (Kruskal–Wallis test, p > 0.05). Indentation rate significantly affected compressive strain (Kruskal–Wallis test, p < 0.05), while creep rate constant was not significantly influenced (p > 0.05). Significance levels are indicated as ns (not significant), *p < 0.05, and **p < 0.01.

### 3.2 Microscale tribological properties of articular cartilage

Microscale tribological properties of the AC surface were evaluated under varying load (Figure 5A) and varying sliding speed conditions (Figure 5B). Significant differences in COF were observed under both conditions. Under varying load conditions, COF ranged 0.001 to 0.036 with CV ranging from 37.53% to 99.02%, showing an overall significant effect (One-way ANOVA, p = 0.0055; Figure 5C). Similarly, under varying sliding speeds, COF ranged 0.002 to 0.047 with CV values ranging from 39.33% to 73.00%, also showing significant differences (One-way ANOVA, p = 0.0127; Figure 5D). COF values decreased with increasing load and increased with increasing sliding speed.

**FIGURE 5:**
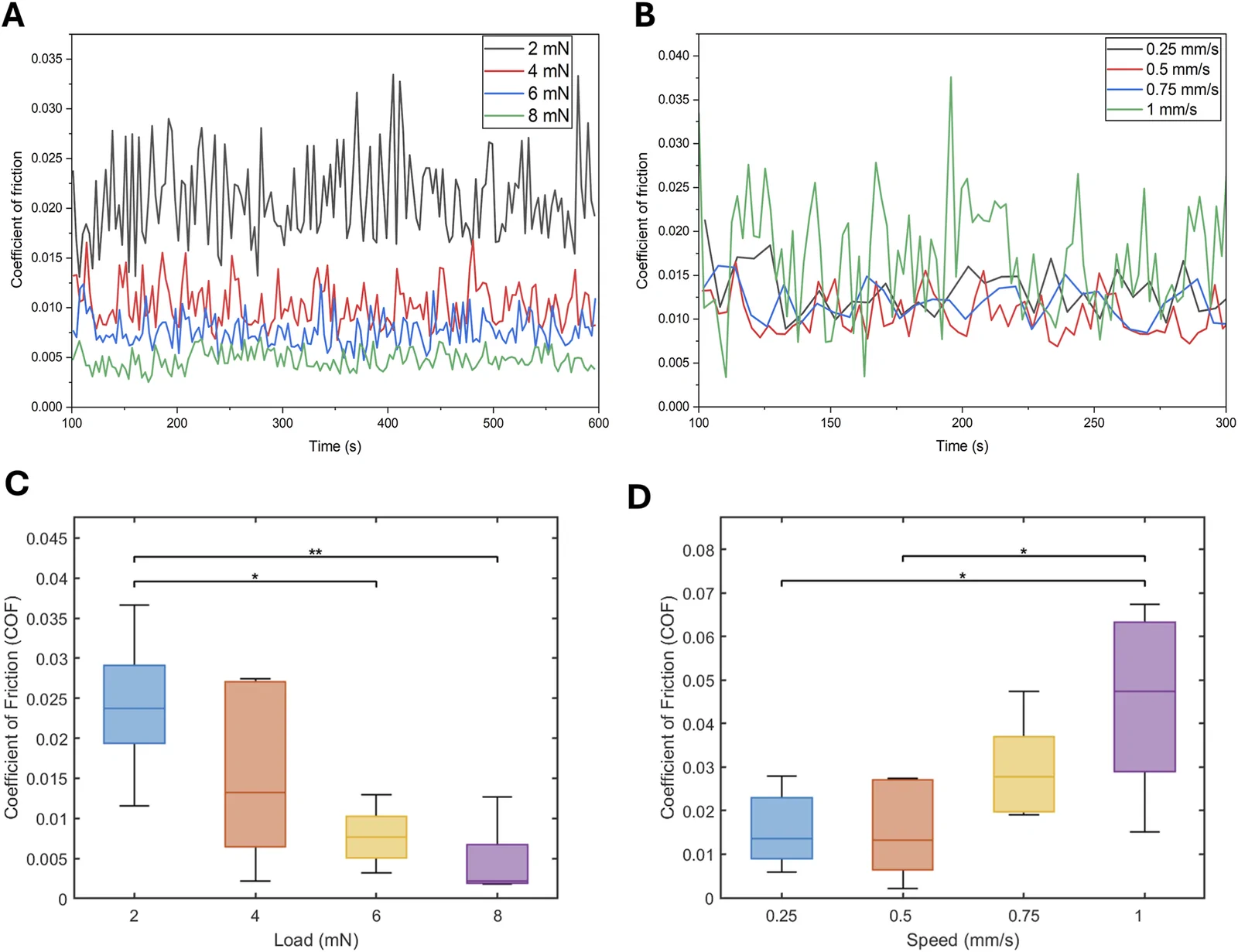
**(A)** The plot of COF v/s time for varying load with constant speed 0.5 mm/s. **(B)** The plot of COF v/s time is plotted for varying speed with constant load 4 mN. **(C)** The plot of COF v/s time is plotted for varying load with constant speed 0.5 mm/s. **(D)** The plot of COF v/s time is plotted for varying speed with constant load 4 mN. Data in **(C,D)** are presented as box plots showing the median, interquartile range, and range. Load had a significant effect on COF (one-way ANOVA, p < 0.01), with COF decreasing with increasing load. Sliding speed also significantly influenced COF (one-way ANOVA, p < 0.05), with COF increasing at higher speeds. Significance levels are indicated as *p < 0.05 and **p < 0.01. (COF: coefficient of friction).

### 3.3 Structural characterization of articular cartilage

#### 3.3.1 Scanning electron microscopy

Qualitative structural characterisation of AC was performed using SEM (surface and cross-section) and histology (cross-section). Macroscopically, the AC surface appeared smooth (Figure 6A) while microscale imaging revealed organized collagen fibril bundles forming the lamina splendens parallel to the surface (Figure 6B). Cross-sectional imaging demonstrated an overall cartilage thickness of 1.297 ± 0.152 mm (Figure 6C). Further, the magnified images of the different zones are shown in Figures 6D–F. Each zone revealed different orientation of the ECM lacunae, where SZ (20% thickness from the surface) (Figure 6D) showed the parallelly oriented ECM lacunae, representing the lamina splendens. In the MZ (30%–70% thickness from the surface) (Figure 6E) and DZ (70%–90% thickness from the surface) (Figure 6F) there was random and radial organisation of ECM respectively. The size of ECM lacunae area was SZ = 97.24 ± 15.70 µm^2^, MZ = 333.70 ± 66.96 µm^2^ and DZ = 735.61 ± 79.43 µm^2^) which increased down the zones. These observations demonstrate qualitative depth-dependent differences in the structural organisation of the cartilage extracellular matrix across the three anatomical zones.

**FIGURE 6:**
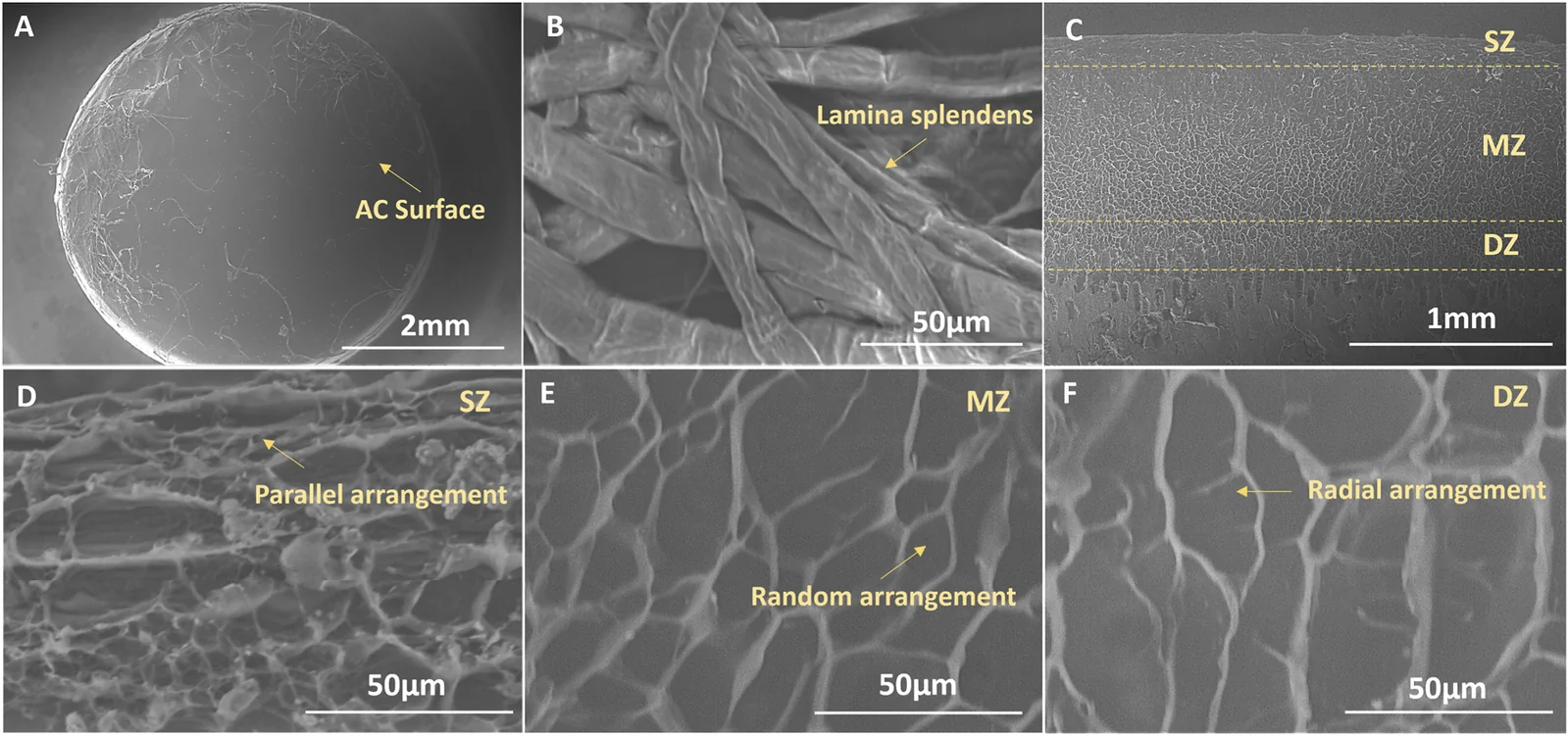
**(A)** The SEM image of the surface of the AC plug. **(B)** The SEM image of the lamina splendens on the surface of the AC showing the SZ. **(C)** The cross-section SEM image of the AC showing three different zones, i.e., SZ, MZ and DZ. **(D)** The SEM image of the SZ showing the parallel arrangement of ECM lacunae. **(E)** The SEM image of the MZ showing the random orientation of the ECM lacunae. **(F)** The SEM image of the DZ showing the radial arrangement of the ECM lacunae. (SEM: scanning electron microscope, SZ: superficial zone, MZ: middle zone, DZ: deep zone, ECM: extracellular matrix).

#### 3.3.2 Histological evaluation

Histological characterization of AC was performed using H & E and SO & FG staining. These images supported clear differentiation of AC constituents across various zones. The distinction of the cellular arrangement was observed using H & E staining (Figure 7A) alongside the magnified images of the different zones of the AC in Figure 7B–SZ, Figure 7C–MZ, Figure 7D–DZ. Similarly, the SO & FG staining of the AC is shown in Figure 7E which highlights the proteoglycan and collagen distribution with red and blue/green colors respectively. Further, the magnified images of the SO & FG from each zone are shown in Figure 7F–SZ, Figure 7G–MZ, and Figure 7H–DZ.

**FIGURE 7:**
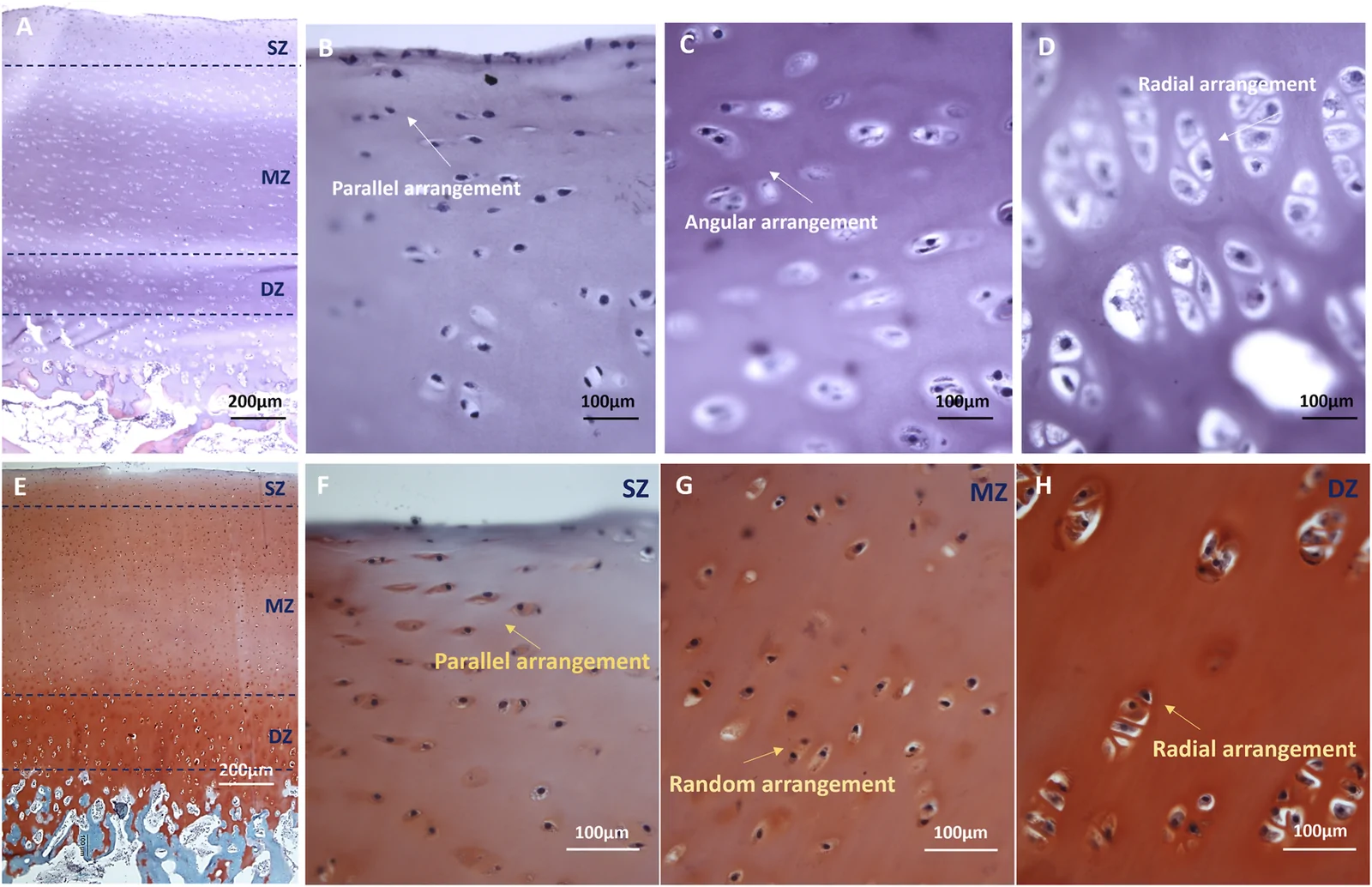
Light microscope images of the AC cross sections stained with Hematoxylin and Eosin stain. **(A)** Cross section **(B)** Superficial zone **(C)** Middle zone **(D)** Deep zone and Safranin O and Fast green stain **(E)** Cross section **(F)** Superficial zone **(G)** Middle zone **(H)** Deep zone.

### 3.4 Quantitative zonal characterization

#### 3.4.1 Histomorphometric analysis

The quantitative characterisation of the AC cartilage surface and the zones were performed using histomorphometry and Raman spectroscopy. In Figure 8A the chondrocyte density significantly varied across the zones (one-way ANOVA, p = 0.0336). The SZ has the higher cell density of 0.0007 ± 0.0001 cells/µm^2^ in comparison to MZ and DZ (both 0.0005 ± 0.0001 cells/µm^2^). This variability was moderate across the zones with CV = 18.02–27.58%. Whereas, in the case of proteoglycan intensity there was also significant difference (one-way ANOVA, p = 0.012) (Figure 8B). However, DZ exhibited highest intensity with 0.0386 ± 0.0197, followed by MZ with 0.0148 ± 0.0015 and the lowest at SZ with 0.0088 ± 0.0028 intensity values. There was highest variability at DZ with CV 51.07% and lowest in the MZ (10.02%) and moderate in SZ (32.20%). This indicates heterogenous matrix distribution across the zones. Finally, the cellular orientation demonstrated highly significant factors using one-way ANOVA with p = 2.31 × 10^−8^. This showed the arrangement of the cells across different zones with respect to their surface with low alignment angles at SZ with 4.90° ± 1.15° (CV = 23.43%). The MZ exhibited greater orientation to the surface with 67.24° ± 4.32° (CV = 6.43%) and the DZ was more perpendicular to the surface with 80.65° ± 8.33° (CV = 10.33%) as shown in Figure 8C.

**FIGURE 8:**
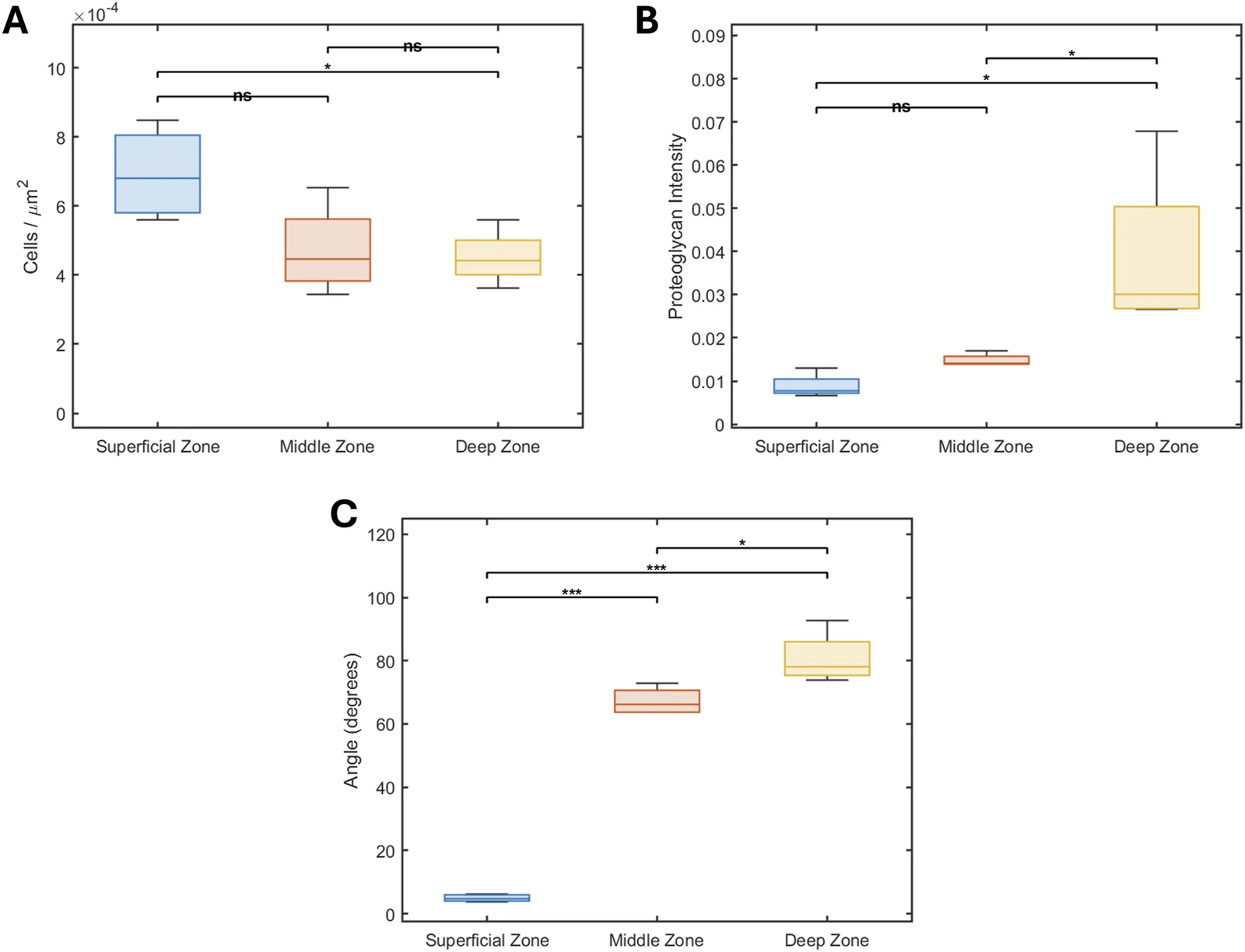
Histomorphometric data from the safranin O and Fast green staining of AC. **(A)** Chondrocyte density (cells/µm^2^) across SZ, MZ and DZ. **(B)** Proteoglycan intensity across SZ, MZ and DZ. **(C)** Cellular orientation across SZ, MZ and DZ. Data are presented as box plots showing the median, interquartile range, and range. One-way ANOVA showed the significance at different zones for chondrocyte density (*p* = 0.0336), proteoglycan intensity (*p* = 0.0121) and cellular orientation (*p* = 2.31 × 10^−8^). Pairwise comparisons were performed using Tukey–Kramer *post hoc* with significance indicated using ns (not significant), *p* < 0.05, and **p < 0.001. (SZ: superficial zone, MZ: middle zone, DZ: deep zone).

#### 3.4.2 Raman spectroscopic analysis

Raman spectroscopic measurements at the cross section (SZ, MZ and DZ) of the AC revealed the distribution of various ECM constituents. The collagen organisation for different zones is shown in Figure 9A, where there is relatively smaller variability across all the zones with CV below 5%. There was no statistical significance (One-way ANOVA p = 0.256) with ratios at SZ (0.976), MZ (1.007) and DZ (0.985). This shows uniform collagen organisation across the zones. Whereas the proteoglycan distribution demonstrated an increasing trend from SZ (0.368), MZ (0.483) and DZ (0.551). However, there was no statistical significance (Kruskal-Wallis, p = 0.3104) shown in Figure 9B.

**FIGURE 9:**
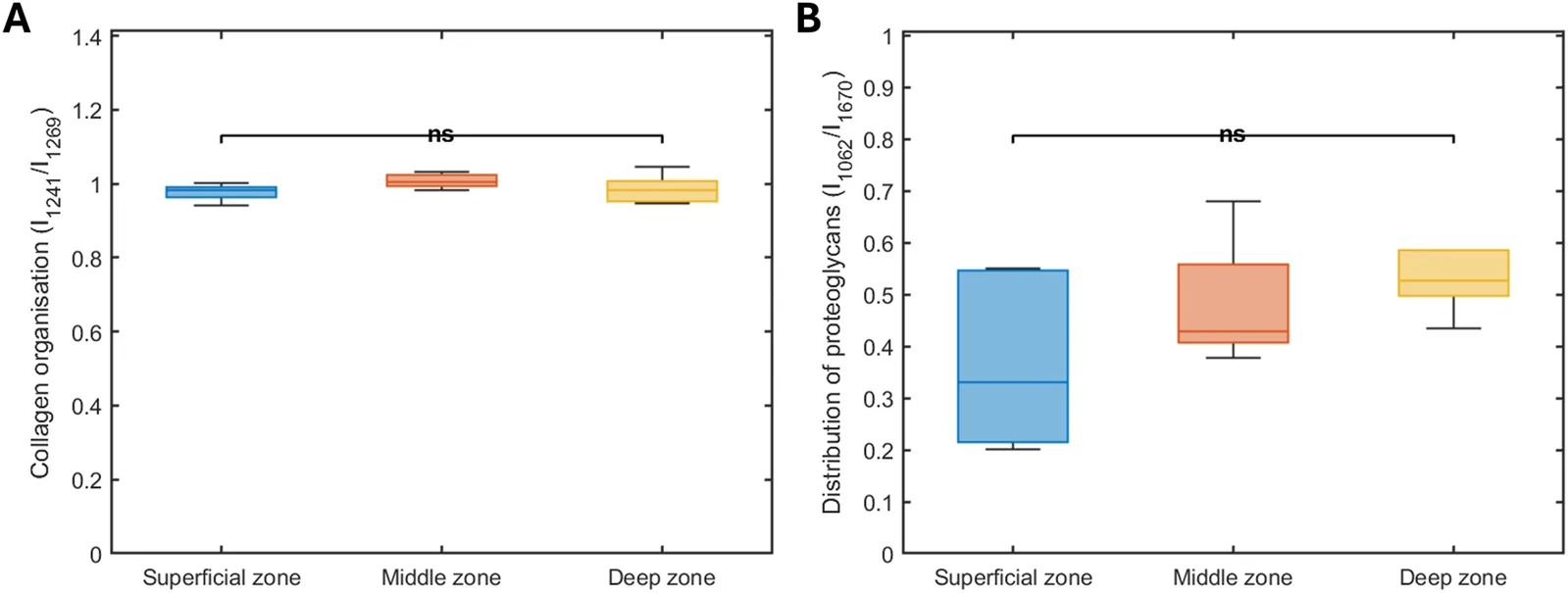
Quantitative Raman spectroscopic data **(A)** and **(B)** are presented as box plots showing the median, interquartile range, and range. One-way ANOVA showed no significant for zonal differences in collagen organisation (*p* = 0.256) and Kruskal–Wallis showed no significant in zonal difference for proteoglycan distribution (*p* = 0.3104). Not significant is denoted as ns. (I: normalised intensity, SZ: superficial zone, MZ: middle zone, DZ: deep zone).

## 4 Discussion

This study investigated the microscale mechano-tribological properties of AC at the surface alongside its structural and biochemical characteristics. The combined methodology enabled mechanical, tribological, histomorphometric, structural and Raman-derived properties to be assessed within the same experimental framework.

The present study revealed a constant effective instantaneous modulus across the surface, suggesting that this response is due to the relatively small Hertzian contact radius (approximately 140–250 µm) confined deformation primarily to the superficial cartilage matrix (Muir et al., 1970). Although AC exhibits heterogeneous, anisotropic, and viscoelastic behaviour, the shallow indentation depths employed in this study limited deformation to the superficial cartilage layer, making the Hertz contact model a reasonable approximation for comparative microscale measurements. The modulus values when tested across the surface of AC revealed minimal load (0.43–0.51 MPa, p = 0.452) and rate (0.38–0.46 MPa, p = 0.621) dependence in contrast to macroscale tests (Loparic et al., 2010; Miller and Morgan, 2010; Park et al., 2004). Similar findings have been reported in previous microscale investigations due to dominant collagen-rich solid matrix stiffness reducing the poroelastic effect (Wahlquist et al., 2017; Nia et al., 2011; Miller and Morgan, 2010). Furthermore, the compressive strain ranged 1.12%–2.18%, p = 0.23 while the creep rate constants ranged 0.02–0.05 s^-1^, p = 0.55 with varying load indicating that the rate of viscoelastic deformation remained largely unchanged despite changes in applied load. The relatively constant creep rate constant suggests that the kinetics of viscoelastic deformation were governed primarily by solid matrix interactions rather than fluid redistribution (Han et al., 2011). However, with increasing indentation rate the compressive strain increased (1.34%–1.88%, p = 0.023) whereas creep rate remained constant (0.02–0.04 s^-1^, p = 0.247) suggesting that loading rate influenced the magnitude of deformation but not the indentation rate at which equilibrium was reached (Islam et al., 2020).

In contrast with the microscale measurements, the mechanical behaviour of the AC at macroscale is mainly governed by poroelastic fluid flow and contributions from deeper cartilage zones. During macroscale cartilage compression, larger contact radii (hundreds of micrometers to millimeters), distribute the load across greater tissue volume. Thereby promoting the interstitial fluid pressurisation supporting up to 70%–90% of applied load, according to biphasic theory (Mak et al., 1987; Mow et al., 1989). This results in higher aggregate modulus (modulus that is due to contribution of both solid and fluid matrix) (∼0.2–1.0 MPa) and greater time-dependent creep behaviour with relaxation occurring over characteristic times of several seconds to minutes. Further, this is influenced by the tissue thickness, permeability and applied load which increases stiffness because of restricted fluid exudation and increased fluid pressure (Krishnan et al., 2004; Ateshian, 2009; Mak et al., 1987; Mow et al., 1989). However, at the microscale, this influence could be minimised by focusing on the near interface solid matrix in SZ which is due to distribution of collagens as lamina splendens.

The microscale tribological properties were evaluated using the changes in the COF measured at the surface. COF decreased significantly with increasing normal load (0.001–0.036, p = 0.005) and were consistent with literature (Park et al., 2004; Chan et al., 2011). Previous studies at the microscale have shown that there is greater confinement of superficial collagen networks entrapping the boundary lubricants on applied load at the asperity level which contributes to lower COF (Park et al., 2004; Chan et al., 2011). In addition, the interstitial fluid pressurisation is minimised at the microscale contacts due to the contact areas being smaller leading to solid matrix bearing the load (Chan et al., 2011; Desrochers et al., 2013; Soltz and Ateshian, 1998; Krishnan et al., 2004). Increasing the applied load enhances the interfacial conformity between contacting asperities, reducing microscale COF (Chan et al., 2011; Chatterjee et al., 2020). In contrast to the load-dependent trend, the COF increased significantly with sliding speed (0.002–0.047, p = 0.012). This behaviour suggests dominant boundary lubrication mechanisms due to entrapment of the boundary lubricants (Klein, 2006; Neu et al., 2008). The observed frictional response may also be attributed to the greater adhesion at the microscale leading to higher stick-slip motions as reported in the previous studies (Drummond et al., 2003; Urbakh et al., 2004; Crockett et al., 2005). In macroscale studies, COF typically decreases with sliding speed because of enhanced hydrodynamic lubrication and fluid pressurization (Ateshian, 2009; Forster and Fisher, 1999). These fluid-related effects are minimized at the microscale, allowing direct assessment of the intrinsic tribological behaviour of the cartilage solid matrix.

Qualitative SEM and histological analyses revealed distinct collagen and extracellular matrix organization across the articular cartilage surface and zonal cross-sections (SZ, MZ, and DZ). The SZ exhibited highly organized collagen fibrils and ECM lacunae, which may contribute to the uniform microscale mechanical, and tribological behaviour observed at the cartilage surface. Previous studies have reported that microscale collagen damage during early to moderate osteoarthritis reduces elastic modulus by 30%–70% (Darling et al., 2010). Further, the collagen damage also increases COF by up to 50% because of impaired boundary lubricant retention (Caligaris and Ateshian, 2008).

Quantitative histomorphometric and Raman analyses revealed distinct zonal variations in the structural and biochemical organization of AC. Cellular density was significantly higher at the SZ in comparison to the other zones (p = 0.033). Whereas the proteoglycan intensity (p = 0.012) and the orientation of cells (p = 2.31 × 10^−8^) with respect to the surface were significantly greater at DZ. Similarly, the collagen organisation showed no significant zonal variation (p = 0.256), while proteoglycan distribution was higher in the DZ but not statistically significant (p = 0.3104). These zonal characteristics contribute to the behaviour of healthy AC. The collagen-rich SZ, with higher cellular density and organized collagen fibrils, provides tensile strength, shear resistance, and boundary lubrication support (Krishnan et al., 2004). Whereas proteoglycan-rich DZ with vertically aligned collagen fibrils contributes to compressive load support and interstitial fluid pressurization at the macroscale (Mow et al., 1980). Therefore, these structural and compositional characteristics of the SZ provide important context for interpreting microscale measurements performed near the cartilage surface (Mansfield et al., 2015; Darling et al., 2004).

Although this study provided insight into the microscale organization and mechano-tribological behaviour of AC. There are several limitations that should be acknowledged. Firstly, the investigations were limited to the microscale and short-term mechanical and tribological behaviors and did not extend to long duration macroscale experiments. Secondly, the tribological experiments were performed under phosphate-buffered saline lubrication and did not incorporate the synovial fluid as in physiological conditions. Inclusion of synovial fluid constituents such as hyaluronan, lubricin, and phospholipids may provide improved understanding of boundary and mixed lubrication mechanisms. The present study characterises mechanical, tribological and biochemical properties within healthy bovine cartilage but was not designed to establish relationships between individual ECM constituents and functional properties. Controlled selection of specific matrix constituents, together with larger independent sample groups, would be required to establish such mechanistic relationships. Furthermore, combining macroscale testing, advanced lubrication assessments, and multiscale modelling may provide a more comprehensive understanding of how microscale ECM architecture governs joint mechanics.

## 5 Conclusion

This study established an integrated framework for assessing microscale mechanical, tribological, structural and biochemical properties of AC. Microscale mechanical behaviour demonstrated relatively consistent effective instantaneous modulus, compressive strain and rate constant across the surface. Whereas tribological behaviour demonstrated significant load and speed-dependent changes in the COF across the surface. Further, SEM, histomorphometry and Raman spectroscopy characterised the distinct structural and compositional organisation of cartilage zonal architecture. Together, these complementary measurements provide a multiscale description of cartilage properties without relying solely on macroscale functional assessment. This experimental framework suggests that microscale testing provides a more direct assessment of intrinsic solid matrix behaviour, which is often masked during macroscale evaluation. This knowledge is critical in osteoarthritic cartilage where solid matrix governs functional deterioration (Elahi et al., 2023; Rajankunte Mahadeshwara et al., 2024). Finally, this study proposes the integration of microscale biological and mechano-tribological methods as an integrated platform that could be used in the future to assess disease-related changes and evaluate potential lubrication-based and regenerative cartilage therapeutic strategies.

## Data Availability

The original contributions presented in the study are included in the article/Supplementary Material, further inquiries can be directed to the corresponding authors.
