# Supplementary material for: A framework to assess mechano-tribological, structural and biochemical properties of articular cartilage
Source: Front Bioeng Biotechnol. 2026 Oct 1;14:1945270. doi: 10.3389/fbioe.2026.1945270 (PMC13668855; doi:10.3389/fbioe.2026.1945270)
Supplement: Supplementary file 1 [file Table1.docx]

Supplementary Material

# 1 Mechanical Properties – Indentation data

Table 1. Microscale Effective Instantaneous Modulus of Articular Cartilage under Varying Normal Loads and Indentation Rates

| Parameter | Condition | Mean ± SD (MPa) | CV (%) | Overall *p*-value |
| --- | --- | --- | --- | --- |
| Effective Instantaneous Modulus (MPa) | **Load (mN)** |  |  | **One-way ANOVA**  **0.4524** |
|  | 2 | 0.459 ± 0.093 | 20.30 |  |
|  | 4 | 0.385 ± 0.025 | 6.39 |  |
|  | 6 | 0.468 ± 0.113 | 24.03 |  |
|  | 8 | 0.464 ± 0.071 | 15.33 |  |
| Effective Instantaneous Modulus (MPa) | **Indentation Rate (µm/s)** |  |  | **One-way ANOVA**  **0.6212** |
|  | 2 | 0.516 ± 0.121 | 23.52 |  |
|  | 4 | 0.487 ± 0.095 | 19.51 |  |
|  | 6 | 0.432 ± 0.067 | 15.56 |  |
|  | 8 | 0.464 ± 0.071 | 15.33 |  |

Table 2. Microscale Compressive Strain and creep rate constant (s^-1^) of Articular Cartilage under Varying Normal Loads and Indentation Rates

| Parameter | Condition | Mean ± SD | CV (%) | Overall *p*-value |
| --- | --- | --- | --- | --- |
| Compressive Strain (%) | **Load (mN)** |  |  | **Kruskal-Wallis 0.2309** |
|  | 2 | 1.143 ± 0.038 | 3.31 |  |
|  | 4 | 1.707 ± 0.058 | 3.38 |  |
|  | 6 | 1.353 ± 0.225 | 16.61 |  |
|  | 8 | 1.757 ± 0.709 | 40.38 |  |
| Creep rate constant (s^-1^) | **Load (mN)** |  |  | **Kruskal-Wallis 0.5551** |
|  | 2 | 0.026 ± 0.017 | 67.39 |  |
|  | 4 | 0.106 ± 0.118 | 111.61 |  |
|  | 6 | 0.037 ± 0.021 | 56.51 |  |
|  | 8 | 0.046 ± 0.021 | 46.57 |  |
| Compressive Strain (%) | **Indentation Rate (µm/s)** |  |  | **Kruskal-Wallis 0.02339** |
|  | 2 | 0.918 ± 0.079 | 8.65 |  |
|  | 4 | 1.353 ± 0.225 | 16.61 |  |
|  | 6 | 1.507 ± 0.104 | 6.91 |  |
|  | 8 | 1.983 ± 0.046 | 2.33 |  |
| Creep rate constant (s^-1^) | **Indentation Rate (µm/s)** |  |  | **Kruskal-Wallis**  **0.2478** |
|  | 2 | 0.058 ± 0.003 | 4.72 |  |
|  | 4 | 0.037 ± 0.021 | 56.51 |  |
|  | 6 | 0.051 ± 0.017 | 32.51 |  |
|  | 8 | 0.039 ± 0.013 | 33.42 |  |

# 2 **Tribological properties**

Table 3. Microscale Coefficient of Friction of Articular Cartilage under Varying Normal Loads and Sliding Speeds

| Parameter | Condition | Mean ± SD | CV (%) | Overall *p*-value |
| --- | --- | --- | --- | --- |
| Coefficient of Friction (COF) | **Load (mN)** |  |  | **One-way ANOVA 0.005582** |
|  | 2 | 0.0241 ± 0.0090 | 37.53 |  |
|  | 4 | 0.0155 ± 0.0113 | 73.00 |  |
|  | 6 | 0.0078 ± 0.0037 | 47.57 |  |
|  | 8 | 0.0047 ± 0.0046 | 99.02 |  |
| Coefficient of Friction (COF) | **Sliding Speed (mm/s)** |  |  | **One-way ANOVA 0.01279** |
|  | 0.25 | 0.0157 ± 0.0088 | 56.22 |  |
|  | 0.50 | 0.0155 ± 0.0113 | 73.00 |  |
|  | 0.75 | 0.0296 ± 0.0116 | 39.33 |  |
|  | 1.00 | 0.0451 ± 0.0213 | 47.30 |  |

3. Histomorphometry quantification stats

Table 4. Histomorphometric Characterization of Articular Cartilage across Superficial, Middle, and Deep Zones

| Parameter | Condition | Mean ± SD | CV (%) | Overall *p*-value |
| --- | --- | --- | --- | --- |
| Proteoglycan Intensity | **Zone** |  |  | **One-way ANOVA 0.0121** |
|  | Superficial Zone | 0.0088 ± 0.0028 | 32.20 |  |
|  | Middle Zone | 0.0148 ± 0.0015 | 10.02 |  |
|  | Deep Zone | 0.0386 ± 0.0197 | 51.07 |  |
| Collagen Orientation (°) | **Zone** |  |  | **One-way ANOVA**  **2.31 × 10⁻⁸** |
|  | Superficial Zone | 4.9035 ± 1.1488 | 23.43 |  |
|  | Middle Zone | 67.2431 ± 4.3234 | 6.43 |  |
|  | Deep Zone | 80.6499 ± 8.3284 | 10.33 |  |
| Chondrocyte Density (cells/µm²) | **Zone** |  |  | **One-way ANOVA 0.0336** |
|  | Superficial Zone | 0.0007 ± 0.0001 | 19.64 |  |
|  | Middle Zone | 0.0005 ± 0.0001 | 27.58 |  |
|  | Deep Zone | 0.0005 ± 0.0001 | 18.02 |  |

# 3 Raman Spectroscopy Quantification Stats

Table 5. Raman Spectroscopic Characterization of Collagen Organisation and Proteoglycan Distribution across Articular Cartilage Zones

| Parameter | Condition | Mean ± SD | CV (%) | Overall *p*-value |
| --- | --- | --- | --- | --- |
| Collagen Organisation (I₁₂₄₁/I₁₂₆₉) | **Zone** |  |  | **One-way ANOVA 0.2561** |
|  | Superficial Zone | 0.9767 ± 0.0227 | 2.33 |  |
|  | Middle Zone | 1.0075 ± 0.0195 | 1.94 |  |
|  | Deep Zone | 0.9857 ± 0.0395 | 4.01 |  |
| Proteoglycan Distribution (I₁₀₆₂/I₁₆₇₀) | **Zone** |  |  | Kruskal–Wallis **0.3104** |
|  | Superficial Zone | 0.3687 ± 0.1697 | 46.04 |  |
|  | Middle Zone | 0.4835 ± 0.1209 | 25.00 |  |
|  | Deep Zone | 0.5510 ± 0.1163 | 21.10 |  |
